# Formaldehyde Levels and the Indoor Air Quality of an Anatomy Dissection Hall with Different Ventilation Setups

**DOI:** 10.1177/11786302241301590

**Published:** 2024-11-26

**Authors:** Ganesh Handady, Anne Dsouza, Vanishri Nayak, Joseph Abraham

**Affiliations:** Department of Anatomy, Kasturba Medical College, Manipal, Manipal Academy of Higher Education, Manipal, India

**Keywords:** Formaldehyde, ventilation system, anatomy, air quality

## Abstract

During anatomy dissection, the release of formaldehyde (FA) from cadavers and embalming fluids can negatively affect the well-being of students and staff. The exposure of students, staff, and technicians to FA in the dissection hall is a concern. To address this issue, a study measured the FA and air quality (CO_2_ and Total Volatile Organic Compounds- TVOC) with different ventilation setups: natural, fan-based, and air-conditioned. The FA levels and the indoor air quality at the breathing zone were estimated using an air conditioning (AC), fan-based, and naturally ventilated setup. The FA, CO_2_, and TVOC levels were calculated at the cadavers’ head and toe ends, in the pathway, and between the dissection tables. The FA, CO_2_, and TVOC levels were higher near the cadaver and lower in the pathway and between the tables, regardless of the type of ventilation used. Fan-based ventilation had the lowest mean FA, CO_2_, and TVOC levels compared to AC and natural ventilation. However, there was no significant difference in these levels between the ventilation types, except for the toe-ends of the cadavers (*P* < .05), where the toe-end farther from the AC vents had higher levels. The study suggests that areas away from the source of ventilation in the anatomy dissection hall are at risk of having lower air quality. Therefore, in addition to selecting an appropriate ventilation system, placing the donor bodies near the source of ventilation would help optimize FA levels and improve indoor air quality for better working conditions suitable for students and staff.

## Plain language summary

In anatomy labs, the chemicals used to preserve bodies can be harmful to the people working there. A study looked at how different ways of getting fresh air into the lab could help. We found that using fans to bring in fresh air resulted in the lowest levels of harmful chemicals. The study suggests that placing the bodies near the source of fresh air can help make the air safer for students and lab staff.

## Introduction

Human cadaveric dissection is essential for learning anatomy, either through demonstrating the specimens or through the dissection of donor bodies. Though new teaching methods (like using models, virtual reality, etc.) exist, donor bodies play a significant role in teaching anatomy.^1^ For the preservation of the donor bodies, durable (or long-lasting) and cost-effective formaldehyde (FA) is used as an embalming solution for the donor bodies.^2^

FA is a colorless, strong-smelling gas readily soluble in water. It is commonly used as a 37% aqueous solution, formalin, to preserve cadavers in medical schools. The sake of using formalin is its cost-effectiveness, preservation properties, availability, and suitable fixation properties.^2^ Formalin is used for its long-lasting feature and non-degenerative capacity.^3^ The solution is routinely injected into the femoral or the carotid artery. Formalin also has bactericidal, fungicidal, and insecticidal properties, and it is widely used as a restoring and conserving agent; it has good disinfecting features and thereby blocks the entry of decomposers.^2^ It is a productive fixative with rapid perforation of tissues and long-term conservative features.^1^

Apart from its preservative properties, FA has several adverse health effects. During anatomy classes, evaporation of FA from donor bodies and embalming fluids could adversely affect students and staff. The students, staff, and technicians in the dissection hall are frequently exposed to FA.^4^ Short-term exposure may cause itchiness. Long-term exposure has been linked to changes in liver function and carcinoma.^5^ The other symptoms are undesirable odor, profuse lacrimation, and skin issues such as skin dryness, eczema, allergic contact dermatitis, respiratory tract infection, and bronchial asthma.^6^ General tiredness, burning eyes, and nose are also reported.^7^ Exposure to FA has a wide range of adverse effects on health, ranging from allergic reactions to genetic damage.^8^ It is also said to be carcinogenic.^9^ Cancer risk assessment in anatomy laboratories with FA exposure is estimated to be several 1000 times higher than the limit recommended by the EPA (10^−6^).^10^

There are several standards set for the permissible limits of FA levels. The short exposure limit of 1ppm (1.2 mg/m^3^) for 30 minutes is the recommended exposure limit (REL) by The National Institute for Occupational Safety and Health (NIOSH).^11^ The permissible exposure limit (PEL) is 2 ppm, which is the maximum exposure limit during a 15-minute duration, according to the Occupational Safety Health Administration (OSHA).^12^ However, the World Health Organization (WHO) has provided a short-term (30-minute) exposure limit of 0.1 mg/m^3^.^13^

The Sustainable Development Goal (SGD) 3.9 emphasizes reducing deaths and illnesses produced by hazardous chemicals and air.^14^ NIOSH strongly recommends the evaluation of FA and indoor air quality assessment to protect workers exposed to dangerous chemicals. Additionally, it emphasizes frequent checks of airflow at the workplace.^11^ National Medical Council (NMC), India, had laid down guidelines on the dimensions and the requirements for the anatomy dissection halls at medical schools.^15^ Therefore, achieving a healthy indoor environment inside the dissection hall is imperative for the safety of the students and the staff.

The ventilation set-ups and the floor area of the dissection hall influence the FA levels and the indoor air quality. FA levels monitored using a fan-based ventilation set-up have shown remarkably higher values, emphasizing the need for an effective ventilation set-up and a larger floor area.^16^ A local exhaust ventilation system has proven the most effective.^17^ However, the effectiveness of such a setup is not well understood for an anatomy dissection hall with a large number of dissection tables and cadavers operational simultaneously. A computational fluid dynamics (CFD) approach revealed that an AC-based ventilation setup has benefits in optimizing FA levels compared to a simple fan-based setup.^18^ However, evaluation of indoor air quality in a realistic set-up would be required to evaluate the indoor environment of the anatomy hall. The distribution of FA levels is influenced by the airflow rate and direction.^13^

People spend over 87% of their time indoors, in buildings contaminated with various chemicals that can adversely affect health even after brief contact. The most often detected indoor air pollutants are volatile organic compounds (VOCs), carbon monoxide (CO), carbon dioxide (CO_2_), nitrogen dioxide (NO_2_), and particulate matter with a diameter of less than 2.5 μm (PM2.5).^19^ The recommended permissible value of total volatile organic compound (TVOC) is 0.3 mg/m^3^.^20^ VOCs, which include acetaldehyde and formaldehyde, are responsible for numerous chemical sensitivities and sick-building syndrome.^21^ VOC inhalation is linked to several harmful health outcomes. Certain VOCs, like formaldehyde and, propylene glycol and glycol ethers (PG), react strongly with the mucous membrane and respiratory tract epithelium.^22^ The recommended permissible value of indoor carbon dioxide should not exceed 1000 ppm.^23^ CO_2_ is a toxin, and it is also a cause of asphyxiation due to hypoxia. It has been demonstrated that respiratory arrest occurs within 1 minute at high concentrations, and unconsciousness occurs practically instantly.^24^

It is uncommon for the literature to evaluate the comprehensive air quality of an anatomy hall in terms of FA levels, carbon dioxide (CO_2_), and total volatile organic compounds (TVOC). Furthermore, the effect of frequently utilized ventilation systems, including air-conditioning (AC) and fan-based ventilation, on FA distribution is not commonly assessed.

To ensure a safe and healthy environment in the anatomy hall, evaluating the levels of FA and overall indoor air quality is essential. This will help identify areas where hazardous compounds may be present and determine how ventilation systems can mitigate their impact. By analyzing FA levels and indoor air quality in different hall locations with varying ventilation setups, this study aims to provide information that can be used to identify the places at risk with higher FA levels and poor air quality.

### Aim and objectives

This study aimed to estimate the FA levels and evaluate the indoor air quality (CO_2_ and TVOC levels) at different locations of an anatomy dissection hall and to find out how these values are influenced by the most commonly operating ventilation systems (natural, fan-based, and air-conditioned (AC).

The objectives were,

1. Estimation of FA levels at different locations (head-end and toe-end of the donor bodies, between tables and pathway) in an anatomy dissection hall
2. Comparison of FA levels with different ventilation systems (AC, fan-based and natural) in an anatomy dissection hall
3. Comparison of TVOC levels and CO_2_ with different ventilation setups and different locations of an anatomy dissection hall

## Materials and Methods

This cross-sectional study was conducted at Kasturba Medical College, Manipal, India, from January to March 2022. The Institutional Ethical Committee of Kasturba Medical College approved the study (IEC no. 614/2021). All methods were performed according to the relevant guidelines and regulations. Written informed consent was obtained during the procurement of cadavers through the voluntary body donation program. The donor, as well as the next of kin, consented to utilize cadavers for research, and medical education.

### Description of the dissection hall involved

The dissection hall used for the current study is designed to accommodate 250 students per the NMC guidelines.^15^ The basic dimensions and infrastructure are described in Table 1.

**Table 1.** Description of the dissection hall used for the study.

Total floor area	925 m^2^
Ventilation options available	AC, fan, and natural ventilation
AC	The centralized operating capacity of 17,000 cfm, vents situated at the height of 2.03 m from the floor level
Number of ceiling fans	40
Distance between the fans	0.96 m
Number of windows	18, 0.52 m × 0.75 m in dimension
Door	One main door
Number of cadavers utilized at a single time	10

There were 10 dissection tables placed about 1.68 m apart from each other. Ten donor bodies were used at a time for 1 academic year. The donor bodies were procured through the institute’s voluntary body donation program with written informed consent from the donor and the next of kin for their utilization for research and education. The donor bodies were washed in water before being placed on the tables. Seven tables were continuous with distance in the dissection hall, and the other 3 were placed apart on the other side of the central pathway. Opposite every table were 2 windows 1.2 m from the toe end of the donor bodies. The AC vents were situated close to the head end at a distance of 0.96 m. 40 ceiling fans were fixed at a height of 3.5 m from the floor level.

**Figure 1.**
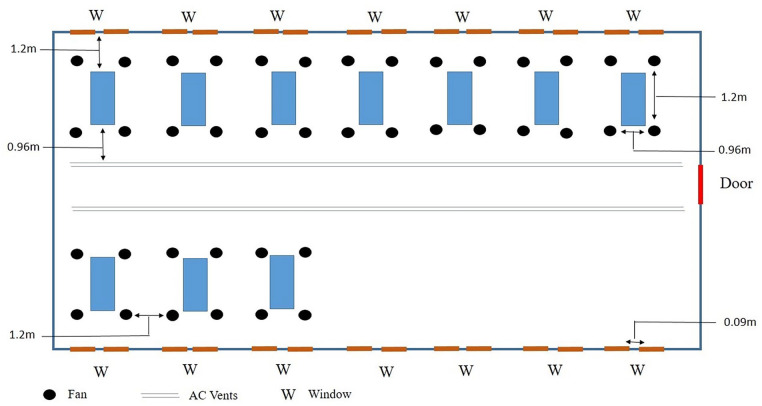
A 2-dimensional pictorial representation of the dissection hall.

Figure 1 provides a 2-dimensional pictorial representation of the dissection hall.

### Description of the embalming fluid used

The embalming fluid at our institution constitutes 50% formalin (the composition is 37% formaldehyde and water in a 1:1 ratio). Arterial embalming is the preferred choice, where the femoral or carotid artery is chosen for injecting the embalming fluid. After embalming, the cadavers are immersed in 50% formalin (the same composition as the embalming fluid).

### A detailed description of formaldehyde estimation and indoor air quality assessment

The students and the staff are involved in dissection activity for 2 hours daily, 4 days a week. Hence, the short-term exposure limit by the WHO was considered the standard for the present study.^25^ InkbirdPlus air quality monitor (Shenzhen, Guangdong, China) was used to measure CO2, FA, and TVOC. It has a measurement range of 0.000 to 1.000 mg/m^3^ of FA, 350 to 2000 ppm of CO_2_, and 0.000 to 2.000 mg/m^3^ of TVOC.

**Figure 2.**
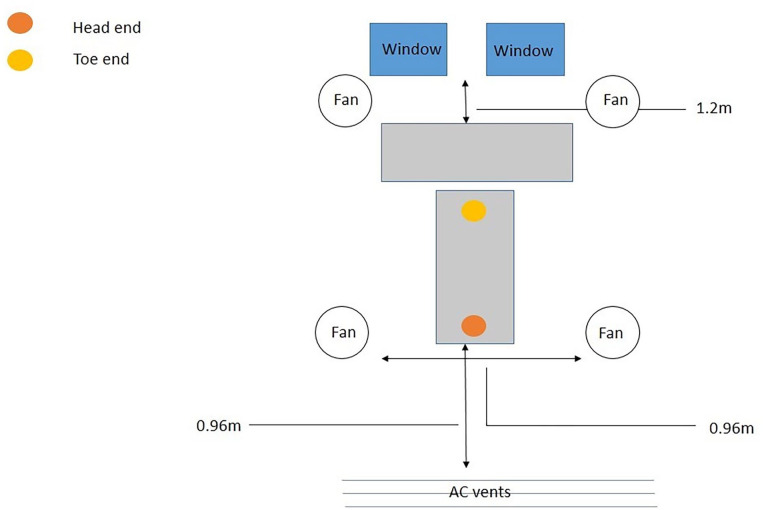
Pictorial representation of the placement of 1 cadaver on a dissection table.

Ten tables were set, each containing 1 formalin embalmed donor body for regular dissection classes. The head end of the donor body is placed near the AC vents, and the toe ends near the windows (Figure 2).

**Figure 3.**
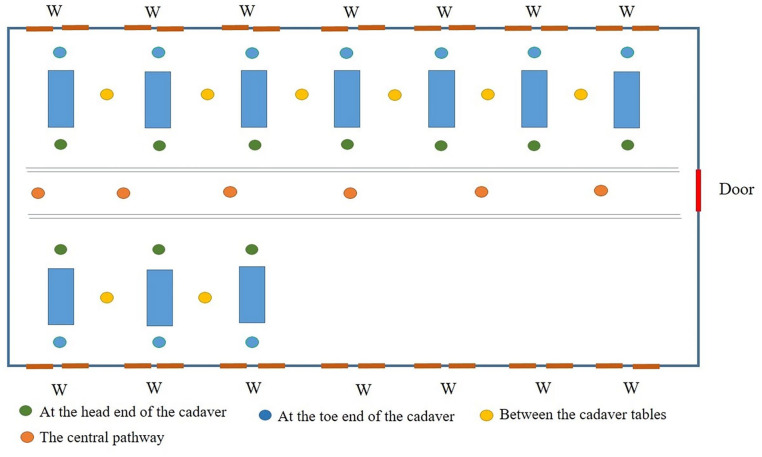
Two-dimensional pictorial representation of the points at which the FA levels and the air quality readings were measured.

The FA levels and the indoor air quality at the breathing zone were estimated using an AC, fan-based, and naturally ventilated setup. All 3 types of ventilation were performed on different days with the same dissection. When the AC was operational, all windows were closed, and all the fans were switched off. Moreover, the naturally ventilated setup had all fans, AC switched off, and windows opened. AC was switched off in the fan-based setup, and the windows were kept open. With these 3 different types of ventilation setups, FA levels and the indoor air quality (CO_2_ and TVOC) were measured at the locations (or points) such as the head end (10 points) and the toe end of the donor bodies (10 points), between 2 the dissection tables (8 points) and in the central pathway, (6 points). The locations are represented in Figure 3. The readings were taken at the breathing zone, that is, at 3 feet (0.914 m) from the floor level. At first, the donor bodies were taken out from the formalin tanks, washed with tap water, and placed on the dissection tables. The ventilation setup was made operational at the same time. After 30 minutes, the values were measured at the defined points with a time interval of 15 minutes between 2 points. Each time before measuring the value, the instrument was switched on. Automatically, the instrument calibrates to zero and then starts readings. It would take 1 to 2 minutes to obtain a stable value. This value was taken as the final value.

During the study period, the donor bodies were utilized to dissection the head, neck, and abdominal regions. The abdomen was dissected, followed by the head and neck. The whole body was kept on the table, and only the head, neck and abdominal regions were exposed while dissecting specific regions. The readings are taken for 4 consecutive working days (Monday to Thursday) for each ventilation setup. All the levels were measured in the absence of students.

The Institutional Ethical Committee of Kasturba Medical College approved the study (IEC no. 614/2021).

### Statistical analysis

Mean and standard deviations were calculated for each variable. The values measured at different points for each ventilation setup were compared using 1-way ANOVA. The values at each end were compared using 1-way ANOVA with the 3 different ventilation setups. Furthermore, Tukey’s Multiple Comparison Test was used for group-wise comparison. GraphPad Prism 5 software (Boston) was used for the statistical analysis.

## Results

### Comparison of FA levels at different locations

We found that the levels of FA varied depending on the type of ventilation used. With AC, the levels ranged from 0.001 to 0.194 mg/m^3^, while with fans, they ranged from 0.002 to 0.059 mg/m^3^, and with natural ventilation, they ranged from 0.001 to 0.23 mg/m^3^. The fan-based ventilation setup had lower mean FA levels (0.01 ± 0.01 mg/m^3^) compared to the AC (0.04 ± 0.05 mg/m^3^) and natural ventilation (0.02 ± 0.03 mg/m^3^). However, the difference was not statistically significant. Additionally, we noticed that in a single ventilation setup, the FA levels were higher at the head and toe-ends of the cadavers compared to the pathway and between the dissection tables. This information can be found in Table 2.

**Table 2.** Mean, standard deviations and coefficient of variation (CV) of FA levels, TVOC, and CO_2_ at different locations and different ventilation setups.

Formaldehyde levels (mg/m^3^) [recommended permissible value-0.01 mg/m^3^]								
	Head end	CV	Toe end	CV	Between tables	CV	Pathway	CV
AC setup	0.0579 ± 0.0544	94%	0.0722 ± 0.0650	90%	0.0078 ± 0.0054	69%	0.01 ± 0.0086	86%
Fan-based setup	0.0207 ± 0.0203	98%	0.0116 ± 0.0127	109%	0.0048 ± 0.0046	96%	0.013 ± 0.0057	44%
Natural ventilation	0.0175 ± 0.0113	65%	0.0436 ± 0.0673	154%	0.0042 ± 0.0024	57%	0.0053 ± 0.0024	45%
Air quality- TVOC levels (mg/m^3^) [recommended permissible value-0.3 mg/m^3^]								
	Head end	CV	Toe end	CV	Between tables	CV	Pathway	CV
AC setup	0.321 ± 0.303	94%	0.408± 0.407	99%	0.0421± 0.0320	76%	0.0541 ± 0.0494	91%
Fan-based setup	0.119 ± 0.109	92%	0.0698 ± 0.0668	96%	0.0305 ± 0.0208	68%	0.065 ± 0.0434	67%
Natural ventilation	0.093 ± 0.067	72%	0.126 ± 0.093	74%	0.0195 ± 0.0053	27%	0.0178 ± 0.0053	30%
Air quality- CO_2_ levels (ppm) [recommended permissible value-400−1000 ppm]								
	Head end	CV	Toe end	CV	Between tables	CV	Pathway	CV
AC setup	861.7 ± 486.5	56%	999.1 ± 488.9	49%	496.1 ± 60.63	12%	519.33 ± 94.79	18%
Fan-based setup	618.4 ± 195.13	32%	534.4 ± 132.34	25%	458.75 ± 49.076	11%	556.83 ± 60.963	11%
Natural ventilation	614.9 ± 134.64	22%	655.7 ± 158.50	24%	451.125 ± 27.0683	6%	467.333 ± 25.2481	5%

In the AC configuration, there was a notable contrast in the FA levels between the toe-end of the cadavers (0.072 ± 0.065 mg/m^3^) and the tables (0.0078 ± 0.0054 mg/m3) as determined by 1-way ANOVA (*P*-value < .0001). However, in both the fan-based and natural ventilation setups, there was no significant difference in FA levels at various locations, suggesting a relatively even distribution of FA levels throughout the dissection hall.

The levels of FA in the pathway and between the tables remained consistent regardless of the type of ventilation system. However, at the toe end, the FA levels were higher with AC than with fan-based ventilation (*P* = .06) and natural ventilation (*P* < .0001).

**Figure 4.**
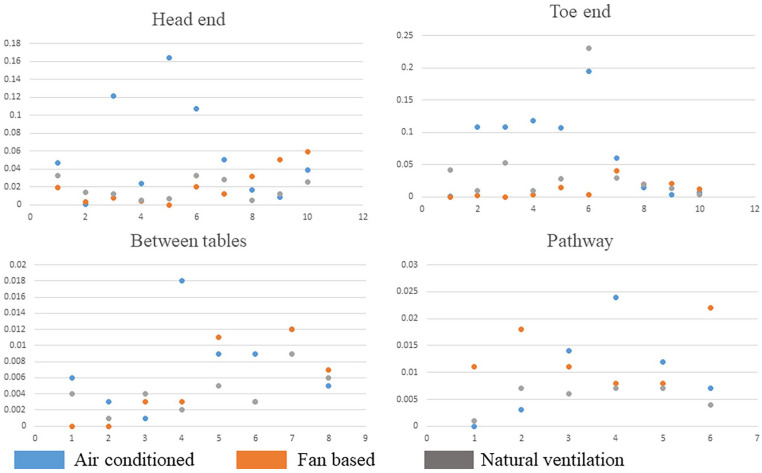
Graphical representation of the distribution of FA levels at different locations of the anatomy hall with different ventilation setups(X-table number, Y-FA values).

Therefore, while comparing the FA levels at different ventilation setups and locations in the dissection hall, the AC setup showed unevenly distributed FA levels compared to the fan-based and natural ventilation setups. Figure 4 indicates the graphical comparison of FA levels with the ventilation systems. Additionally, in the present study, the FA levels were always within the standard limits provided for the short-term exposure limit by NIOSH with all 3 ventilation setups.

### Comparison of TVOC and CO2 levels at different locations

With AC, the CO_2_ levels ranged from 408 to 1839 ppm, while with fans, they ranged from 405 to 971 ppm, and with natural ventilation, they ranged from 409 to 932 ppm. The fan-based ventilation setup had lower mean CO_2_ levels (545.26 ± 138.38 ppm) compared to the AC (773.55 ± 397.5 ppm) and natural ventilation (562.23 ± 139.9 ppm). With AC, the TVOC levels ranged from 0.01 to 1.28 mg/m^3^, while with fans, they ranged from 0.01 to 0.32 mg/m^3^, and with natural ventilation, they ranged from0.01–0.3 mg/m^3^.

According to Table 2, the TVOC and CO_2_ levels were notably elevated at both the head-end and toe-end of the cadavers, in contrast to the pathway and the area between the tables for all 3 ventilation systems. The AC setup had significantly higher CO_**2**_ levels at the toe-end of the cadavers than the fan-based and natural ventilation methods (*P* < .0001). Table 3 compares CO_2_ levels at different locations using the operating ventilation setup.

**Table 3.** Comparison of the CO_2_ levels (ppm) at the different locations of the dissection hall with the ventilation setups.

Location	Comparison between the ventilation setups	Mean difference	95% confidence interval	Significance*
Head end of the cadaver	AC vs Fan-based	243.4	−103.4 to 590.1	No
	AC vs. Natural	246.9	−99.86 to 593.6	No
	Fan-based vs. Natural	3.500	−343.2 to 350.2	No
Toe end of the cadaver	AC vs. Fan-based	464.7	124.7 to 804.7	Yes (*P* < .0001)
	AC vs. Natural	343.4	3.365 to 683.4	Yes (*P* < .05)
	Fan-based vs. Natural	−121.3	−461.3 to 218.7	No
Between tables	AC vs. Fan-based	37.38	−22.73 to 97.48	No
	AC vs. Natural	45.00	−15.11 to 105.1	No
	Fan-based vs. Natural	7.625	−52.48 to 67.73	No
Pathway	AC vs. Fan-based	−37.50	−137.5 to 62.52	No
	AC vs. Natural	52.00	−48.02 to 152.0	No
	Fan-based vs. Natural	89.50	−10.52 to 189.5	No

* Using Tukey’s Multiple Comparison Test.

Regardless of the ventilation setup, the TVOC levels remained consistent between the tables and the pathway. However, at the toe-end of the donor body, the TVOC levels were substantially higher with the AC setup than the natural and fan-based setups (*P* < .001). A comprehensive comparison is given in Table 4.

**Table 4.** Comparison of the TVOC levels (mg/m^3^) at the different locations of the dissection hall with the ventilation setups.

Location	Comparison between the ventilation setups	Mean difference	95% confidence interval	Significance*
Head end of the cadaver	AC vs Fan-based	0.2022	−0.008893 to 0.4133	No
	AC vs Natural	0.2288	0.01771 to 0.4399	Yes (*P* < .05)
	Fan-based vs Natural	0.0266	−0.1845 to 0.2377	No
Toe end of the cadaver	AC vs Fan-based	0.3383	0.06718 to 0.6094	Yes (*P* < .001)
	AC vs Natural	0.2821	0.01098 to 0.5532	Yes (*P* < .001)
	Fan-based vs Natural	−0.05620	−0.3273 to 0.2149	No
Between tables	AC vs Fan-based	0.01163	0.01652 to 0.03977	No
	AC vs Natural	0.02263	−0.005523 to 0.05077	No
	Fan-based vs. Natural	0.0110	−0.01715 to 0.03915	No
Pathway	AC vs. Fan-based	−0.01083	−0.06801 to 0.04634	No
	AC vs Natural	0.03633	−0.02084 to 0.09351	No
	Fan-based vs Natural	0.04717	−0.01001 to 0.1043	No

* Using Tukey’s Multiple Comparison Test.

## Discussion

This study used various ventilation systems to examine the FA, CO2, and TVOC levels in an anatomy dissection hall. We found that the toe-end of the donor bodies had notably higher levels of these compounds in the AC setup, likely due to their distance from the vents. The airflow is typically more confined in an air-conditioned space, particularly in the vicinity of the air vents. As a result, there are certain places where FA may collect more due to uneven air mixing. Since the cadavers’ toe ends are probably farther away from direct airflow sources, there may be stagnation zones where inadequate air circulation causes FA levels to rise. However, there were no significant differences in levels between the fan-based and natural ventilation setups at different points. This might be because both fan-based and natural ventilation systems likely create more homogeneous airflow across the dissection hall than AC, resulting in a relatively even distribution of FA levels.

Research in the anatomy hall evaluations has been documented in the literature, with studies comparing FA levels in different medical colleges. Bhat et al found that the 8-hour TWA for FA was higher in a Private Medical College (ranging from 3.4 to 5 ppm) compared to a Government Medical College (ranging from 1.64 to 2.02 ppm).^5^ In this study, conducted at a private institute, FA levels were within the acceptable limit of 1 ppm for short-term exposure and did not exceed this limit regardless of ventilation setup. It is possible that the difference in FA levels between government and private colleges can be attributed to factors such as the number of cadavers, infrastructure, and the size of the dissection hall. However, the anatomy hall in this study meets NMC guidelines, indicating that the findings can be generalized to other medical institutes.

In the study by Adamović et al, FA levels were estimated at 5 locations inside the Department of Anatomy in Serbia in February, March, and May. Environmental factors, such as humidity and temperature, were also considered. In the cadaver storage room, the FA levels were as high as 1.76 to 2.90 ppm.^10^ Similarly, in the present study, the FA levels were higher around the cadaver compared to the pathway and between tables. However, the present study did not include the storage room for FA level monitoring, considering that inevitably, there are higher FA levels inside the storage room. At the same time, students and staff are exposed to FA in the dissection hall.

Takayanagi et al reported that FA levels ranged from 0.24 to 3.04 ppm during systemic musculoskeletal anatomy and neuroanatomy.^26^ FA levels in the indoor air of the anatomy dissection hall with 35 cadavers at the Faculty of Medicine of Suranaree University were found to be levels between 0.25 and 0.55 ppm.^27^ Our present study measured FA levels during the head, neck, and abdominal wall dissection. We observed a decrease in FA levels in the dissected region, which could be attributed to the smaller surface area compared to the higher values reported by Takayanagi et al.^26^

According to a recent study by Norkaew et al, high levels of FA may contribute to skin-related issues among medical students.^28^ Fortunately, research suggests that implementing a local ventilation system in the anatomy dissection hall can lower FA levels. However, due to the large number of students and cadavers, installing a complete ventilation setup may not be cost-effective. Instead, optimizing the existing ventilation system through regular FA and air quality monitoring could be a viable option.

According to a study by Zuber et al, the AC setup had lower FA levels than the fan-based and natural ventilation setups, as determined by the computational fluid dynamics approach.^18^ However, our present study observed higher levels of FA with the AC setup, although there was no significant difference between the various ventilation setups. This difference could be attributed to the AC vents’ high placement and the direction and circulation of the airflow within the dissection hall.

According to Aung et al, the levels of FA in dissection rooms with a wall-mounted fan as ventilation showed remarkably high values with short-term exposure.^16^ The study suggested that reducing the number of cadavers and increasing the area of the dissection hall could lower FA levels. The present study found that the FA levels were within expected limits for short-term exposure, which may be due to the larger floor area, more ceiling fans, and fewer donor bodies.

Studies have found that fan-based ventilation in dissection halls has resulted in higher formaldehyde (FA) levels.^29,30^ Other reports have shown that personal exposure to FA can be higher than indoor levels when using air conditioning in anatomy halls.^31-33^ In this study, the air conditioning system resulted in higher levels of FA, particularly in the toe-end areas of the donor bodies. Due to their distance from the AC vents, the heavier FA particles were not effectively cleared. However, the FA levels and indoor air quality were better with fan-based ventilation, suggesting that having 4 ceiling fans per cadaver effectively cleared the hazardous compounds near the cadavers.

Previous studies have addressed monitoring formaldehyde (FA) levels in the dissection hall.^5,34^ Gahukar et al conducted a study that evaluated FA levels with and without exhaust fans operating and found higher FA values in the dissection hall.^34^ Table 5 provides a comprehensive overview of indoor FA estimation based on various ventilation setups in previous studies.

**Table 5.** Comparison of indoor FA levels with different dissection halls and ventilation settings.

Authors, Year, and Country	Floor area of the dissection hall (m^2^)	Sampling locations	Type of ventilation setup	Number of cadavers	Average indoor FA levels (ppm)*
Kunugita et al (2004) Japan	1400	Close to the cadavers	• General ventilation • General ventilation with new filters • Novel local ventilation for each dissection table	25	0.76 0.61 0.05
Ohmichi et al (2006) Japan	1125	Center and the four corners of the lab	12 supply diffusor, four air conditioners, eight air return grills	Not mentioned	0.23-1.03
Vohra (2011) Saudi Arabia	450	Center, close to the doors and four corners of the lab	Central air conditioner, four exhaust fans	25	0.68 (week 4) 0.85 (week 10) 0.73 (week 14)
Azari et al (2012) Iran	339	Not specified	• Ventilation (supply-exhaust) system on • Ventilation supply system on • Ventilation system (supply and exhaust) off	Not mentioned	0.306 ± 0.021 0.317 ± 0.026 0.698 ± 0.034
Gahukar et al (2014) India	308	Aisle and corners of the hall; at about 2 feet height At about 5 feet height over dissection tables	Windows with an exhaust fan	16	0.049-0.904
Homwutthiwong and Ongwandee (2017) Thailand	380	Close to the cadaver At the corner Corridor	Windows and four big pedestal fans	14	0.00925-0.01759
Aung et al (2021) Myanmar	Dissection room I, II-11,188m^3^ Dissection room III- V- 9942 m^3^	Center of the dissection hall, six feet above the ground	Ten wall fans in each dissection room	10 (distributed in four dissection rooms)	0.09-1.22
The present study (2022) India	925	Head end, toe-end of the cadavers, between tables and pathway	Air condition Fan-based natural ventilation	10	0.001-0.238 0.002-0.072 0.001-0.246

* Levels in mg/m^3^ are converted into ppm for suitable comparability.

*Note*. Few of the contents of this table were adapted from Table 2 of Aung et al (2021).

This study uses ventilation systems to examine the various dissection hall locations for FA, CO_2_, and TVOC levels. Regardless of the ventilation type, the levels were notably higher around the cadaver, and the air quality was sometimes poor at specific points while using AC ventilation. As formaldehyde in ambient air quickly photo-oxidizes to carbon dioxide, it is imperative to have higher FA and CO_2_ levels near the donor bodies. The present study shows that fan-based ventilation can effectively remove FA, CO_2_, and TVOC from the air around cadavers in a dissection hall. To improve indoor air quality, it is recommended that 4 ceiling fans be installed per donor body. Another effective measure is placing the cadavers near AC vents. However, more research is needed to compare different ventilation setups and assess their impact on indoor air quality in anatomy dissection halls.

### Limitations

The measurements were for a short exposure limit rather than a time-weighted average over an 8-hour interval, which may limit the accuracy in assessing the long-term exposure risks. The furniture placement, like stools and racks, was not considered, which could affect the airflow patterns within the dissection hall, potentially impacting indoor air quality measurements. Additionally, climatic conditions can have an impact on the levels of FA, which were not taken into consideration. The study focused on a single dissection hall, limiting the generalizability of the findings.

### Scope for further research

FA levels and air quality assessment for a longer duration, over a while, can be considered to analyze the climatic influence and effect of ventilation setups. Future research can focus on how natural, with only the window, fan with the windows open, and using AC with the window open setups would influence the FA levels.

## Conclusion

When comparing the area around the tables and the pathway, it was found that the indoor FA levels near the cadaver were higher. This highlights the importance of a reliable ventilation system to clear FA levels. Additionally, the toe-end of the area, which was farther away from the AC vents, showed higher levels of FA, CO_2_, and TVOC. Despite the AC setup being considered the optimal ventilation system, it could not effectively clear the air in this more distant area. The current research indicates that fan-based ventilation results in lower mean FA levels than AC and natural ventilation. To enhance indoor air quality around cadavers in the anatomy dissection hall, it is recommended to position them near AC vents and operate multiple ceiling fans simultaneously. Additionally, selecting an appropriate ventilation system and placing the cadavers near the source of airflow can further optimize FA levels and create a healthier working environment for the students and faculty. Ensuring a safe and healthy workplace should be a top priority for everyone in the anatomy dissection hall.
